# Screening and identification of key biomarkers of papillary renal cell carcinoma by bioinformatic analysis

**DOI:** 10.1371/journal.pone.0254868

**Published:** 2021-08-06

**Authors:** Yingying Xu, Deyang Kong, Zhongtang Li, Lingling Qian, Junchao Li, Chunbo Zou

**Affiliations:** 1 Department of Nephrology, Nanjing University of Traditional Chinese Medicine, Nanjing, Jiangsu, People’s Republic of China; 2 Department of Nephrology, Shenzhen Bao’an District Songgang People’s Hospital, Shenzhen, Guangdong, People’s Republic of China; 3 Department of Pediatrics, Zhongda Hospital, Southeast University, Nanjing, Jiangsu, People’s Republic of China; 4 Department of Vasculocardiology Deparment, Taizhou Clinical Medical College of Nanjing Medical University (Taizhou People’s Hospital), Taizhou, Jiangsu, People’s Republic of China; 5 Department of Nephrology, Taizhou Clinical Medical College of Nanjing Medical University (Taizhou People’s Hospital), Taizhou, Jiangsu, People’s Republic of China; Center for Cancer Research, UNITED STATES

## Abstract

**Background:**

Papillary renal cell carcinoma (PRCC) is the most common type of renal cell carcinoma after clear cell renal cell carcinoma (ccRCC). Its pathological classification is controversial, and its molecular mechanism is poorly understood. Therefore, the identification of key genes and their biological pathways is of great significance to elucidate the molecular mechanisms of PRCC occurrence and progression.

**Methods:**

The PRCC-related datasets GSE7023, GSE48352 and GSE15641 were downloaded from the Gene Expression Omnibus (GEO) database. Differentially expressed genes (DEGs) were identified, and gene ontology (GO) term enrichment analysis and Kyoto Encyclopedia of Genes and Genomes (KEGG) pathway analysis were performed. Cytoscape and STRING were used to construct the protein-protein interaction network (PPI) and perform module analysis to identify hub genes and key pathways. A heatmap of hub genes was constructed using the UCSC cancer genomics browser. Overall survival and recurrence-free survival of patients stratified by the expression levels of hub genes were analysed using Kaplan-Meier Plotter. The online database UALCAN was applied to analyse gene expression based on tissue type, stage, subtype and race.

**Results:**

A total of 214 DEGs, specifically, 205 downregulated genes and 9 upregulated genes, were identified. The DEGs were mainly enriched in angiogenesis, kidney development, oxidation-reduction process, metabolic pathways, etc. The 17 hub genes identified were mainly enriched in the biological processes of angiogenesis, cell adhesion, platelet degranulation, and leukocyte transendothelial migration. Survival analysis showed that EGF, KDR, CXCL12, REN, PECAM1, CDH5, THY1, WT1, PLAU and DCN might be related to the carcinogenesis, metastasis or recurrence of PRCC. UALCAN analysis showed that low expression of PECAM1 and PLAU in PRCC tissues was related to stage, subtype and race.

**Conclusions:**

The DEGs and hub genes identified in the present study provide insight into the specific molecular mechanisms of PRCC occurrence and development and may be potential molecular markers and therapeutic targets for the accurate classification and efficient diagnosis and treatment of PRCC.

## Introduction

Papillary renal cell carcinoma (PRCC) accounts for approximately 15% of renal cancers and is the most common type of renal cell carcinoma after clear cell renal cell carcinoma (ccRCC). Its pathological classification is controversial, and its clinical behaviour is highly variable. Not only can PRCC be divided into histological type 1 and type 2, studies in recent years have shown that type 2 PRCC is heterogeneous; it can be divided into further subtypes according to tumour-related molecular biomarkers, and these subtypes are associated with different clinical processes and prognoses. For example, subgroup C2c contains type 2 PRCC tumours with the CpG island methylator phenotype (CIMP), and the overall survival rate is the lowest in this group [1]. Because the specific molecular pathogenesis of PRCC is poorly understood, there has been no effective targeted therapy for papillary carcinoma in the past. There is accumulating evidence indicating that abnormal gene expression and mutation are related to the development of PRCC. Linehan et al. [2] found through clinical studies that the occurrence of PRCC type 2 was closely related to mutations in the chromatin modification genes SETD2, BAP1 and PBRM1, while mutations in the structural domain of the tyrosine kinase MET were related to type 1 PRCC. In hereditary leiomyomatosis and renal cell carcinoma (HLRCC) (which is an aggressive form of type 2 PRCC), mutation of Fumarate Hydratase (FH) located on chromosome 1 is relatively common [3]. However, there is currently no standard treatment for the disease, and mortality rates for PRCC patients remain high. Therefore, understanding the exact molecular mechanisms involved in the carcinogenesis, metastasis, and recurrence of PRCC and formulating effective diagnosis and treatment strategies are of vital importance to improving the survival rate of patients.

Microarray technology and bioinformatic analysis, important and widely used methods for screening and identification of differentially expressed genes (DEGs) related to disease development, have helped us to explore a wide variety of DEGs involved in PRCC canceration and progression. However, the false positive rate in single microarray analysis is relatively high, and it is difficult to obtain reliable results. Therefore, in this study, 3 mRNA microarray datasets from the Gene Expression Omnibus (GEO) were downloaded and analysed to identify DEGs between PRCC and normal tissues. Subsequently, Gene Ontology (GO) analysis, Kyoto Encyclopedia of Genes and Genomes (KEGG) pathway enrichment analysis and protein-protein interaction (PPI) network construction were used for analysis to help us understand the key genes and pathways involved in carcinogenesis and progression. From this analysis, a total of 214 DEGs and 17 hub genes were selected that may be potential molecular targets and biomarkers of PRCC.

## Materials and methods

### Acquisition of microarray data

The GEO (http://www.ncbi.nlm.nih.gov/geo) database of the National Center for Biotechnology Information (NCBI) is a public functional genomics database that is used to store gene expression datasets, platform information and original series. Three gene expression datasets (GSE7023 [4], GSE48352 and GSE15641 [5]) were downloaded from GEO. GSE7023 is based on the GPL4866 platform (Affymetrix GeneChip Human Genome U133 Plus 2.0 Array) and includes 35 PRCC samples and 12 normal samples. GSE48352 is based on the GPL16311 platform ([HG-U133_Plus_2] Affymetrix Human Genome U133 Plus 2.0 Array) and includes 24 PRCC samples and 8 normal samples. GSE15641 was based on the GPL96 platform ([HG-U133A] Affymetrix Human Genome U133A Array) and includes 11 PRCC samples and 23 normal samples.

### Screening of DEGs

Differential expression analysis was performed for each dataset using the GEO2R online analysis tool provided with the GEO database. The screening criteria for DEGs between PRCC and non-cancerous samples were |log_2_FC (fold change) |> 1 and FDR adjusted P value <0.05. Based on the annotation data in the platform, the probes were converted into the corresponding gene symbol. Probe sets without corresponding gene symbols and duplicate data were removed. Then, the intersection of three datasets was taken to determine the common DEGs, and the online tool Venn Diagram (http://bioinformatics.psb.ugent.be/webtools/Venn/) was used to draw the Venn diagram of the DEGs.

### KEGG and GO enrichment analyses

The Database for Annotation, Visualization and Integrated Discovery (DAVID; https://david.ncifcrf.gov/home.jsp) (version 6.8) was used to conduct GO enrichment analysis of the DEGs. GO includes three categories: biological pathway, cellular component and molecular function. KOBAS 3.0 (http://kobas.cbi.pku.edu.cn/kobas3/annotate/) was used for KEGG pathway enrichment analysis. P<0.05 was considered statistically significant.

### PPI network construction and module analysis

The Search Tool for the Retrieval of Interacting Genes online database (STRING; https://string-db.org (version 11.0)) was used to construct the DEGs PPI network, and the analysis results were visualized with Cytoscape (version 3.6.1); each interaction with a combined score >0.4 was considered statistically significant. The Molecular Complex Detection (MCODE) (version 1.5.1) plug-in in Cytoscape was used to identify the most important modules in the PPI network. The selection criteria were as follows: degree cutoff = 2, node score cutoff = 0.2, K-core = 2 and max depth = 100. The genes in the module were then subjected to GO and KEGG enrichment analyses using DAVID and KOBAS 3.0, respectively.

### Selection and analysis of hub genes

Through the degree algorithm, 17 genes identified to have scores greater than 15 by cytoHubba in Cytoscape were identified as hub genes. These hub genes were analysed and visualized with the Biological Network Gene Oncology (BiNGO) tool (version 3.0.4). The heatmap of the hub genes was constructed using the UCSC cancer genomics browser (http://xena.ucsc.edu/). The associations of the hub genes with overall survival and recurrence-free survival were analysed using Kaplan-Meier Plotter (https://kmplot.com/analysis/). The online database Oncomine (https://www.oncomine.org) was used to analyse heatmaps of PECAM1 and PLAU gene expression in clinical PRCC samples and normal tissues. The online database UALCAN (http://ualcan.path.uab.edu/) was applied to analyse the expression of these two genes based on tissue type (PRCC tissue versus normal tissue), stage, subtype and race.

## Results

### Identification of DEGs in PRCC

**Fig 1:**
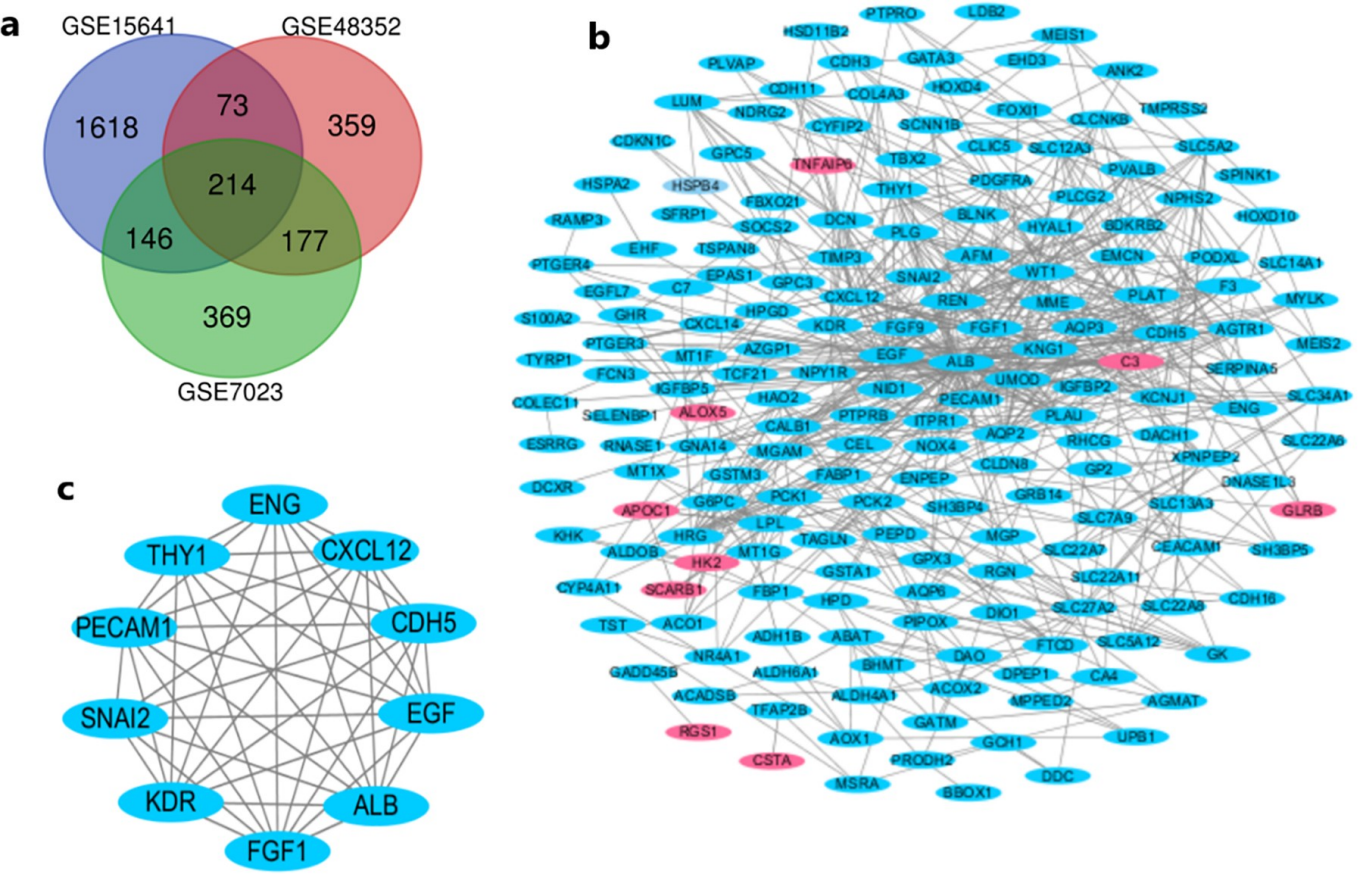
Venn diagram, PPI network and most significant module of the DEGs. (a) DEGs were selected from the mRNA expression profile datasets GSE7023, GSE48352 and GSE15641 with the criteria of |log_2_FC (fold change) |> 1 and FDR adjusted P value <0.05. A total of 214 genes overlapped among the 3 datasets. (b) STRING and Cytoscape were used to construct a PPI network of the DEGs. (c) The MCODE plug-in in Cytoscape was used to identify the most significant module in the PPI network; this module contained 10 nodes and 43 edges. Red represents upregulated genes, and blue represents downregulated genes.

After the microarray results were normalized, DEGs in the three datasets were identified (906 in GSE7023, 823 in GSE48352, and 2051 in GSE15641) (S1 Fig). The overlap between the three datasets contained 214 genes, as shown in the Venn diagram (Fig 1A). The set of 214 overlapping genes contained 9 upregulated genes and 205 downregulated genes between PRCC and normal tissues.

### GO and KEGG enrichment analyses of the DEGs

**Fig 2:**
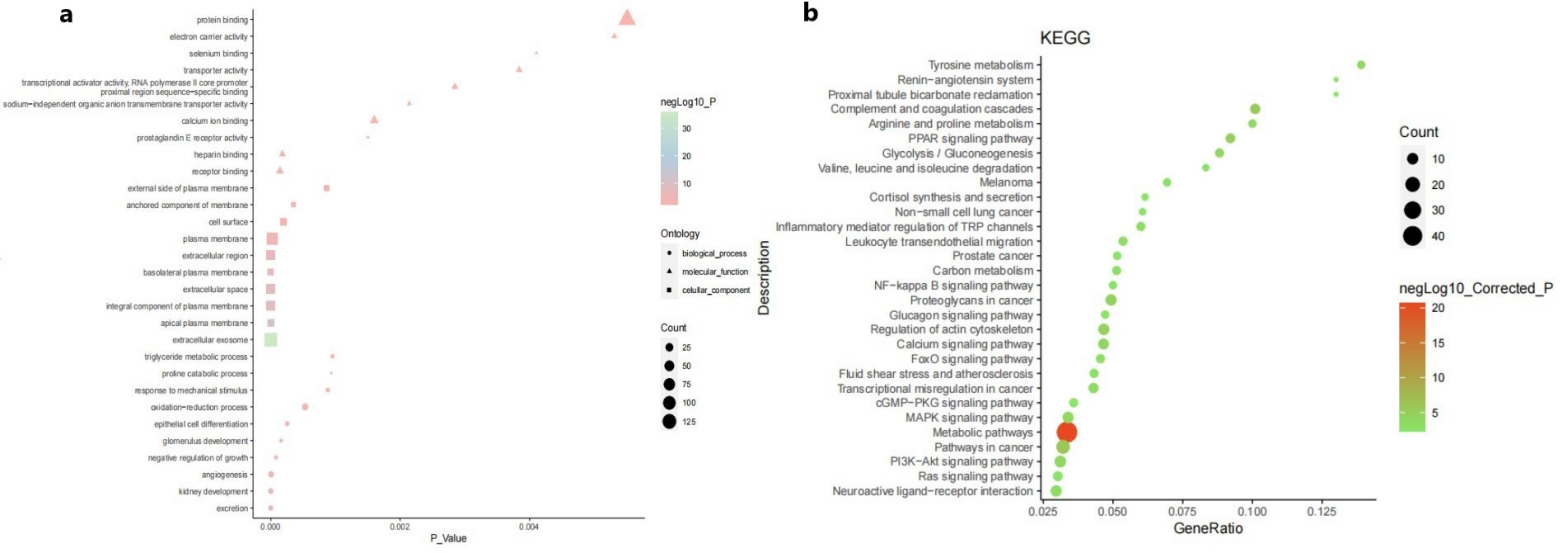
GO enrichment analysis (a) and KEGG pathway analysis (b) of the DEGs in papillary renal cell carcinoma. GO, Gene Ontology; KEGG, Kyoto Encyclopedia of Genes and Genomes.

GO and KEGG enrichment analyses of the DEGs were performed using DAVID and KOBAS 3.0, respectively. The GO analysis results indicated that in terms of biological process, the DEGs mainly participated in excretion, kidney development, angiogenesis, negative regulation of growth, epithelial cell differentiation and oxidation reduction processes. In terms of molecular function, DEGs were mainly associated with receptor binding, heparin binding, prostaglandin E receptor activity, calcium ion binding, sodium-independent organic anion transmembrane transporter activity and transcriptional activator activity. In the cellular component category, the DEGs were mainly enriched in the terms extracellular exosome, apical plasma membrane, integral component of plasma membrane, extracellular region, and cell surface (Fig 2A). KEGG pathway analysis showed that the DEGs were mainly enriched in the following pathways: metabolic pathways, pathways in cancer, complement and coagulation cascades, proteoglycans in cancer and PPAR signaling pathway (Fig 2B).

### PPI network construction and module analysis

**Fig 3:**
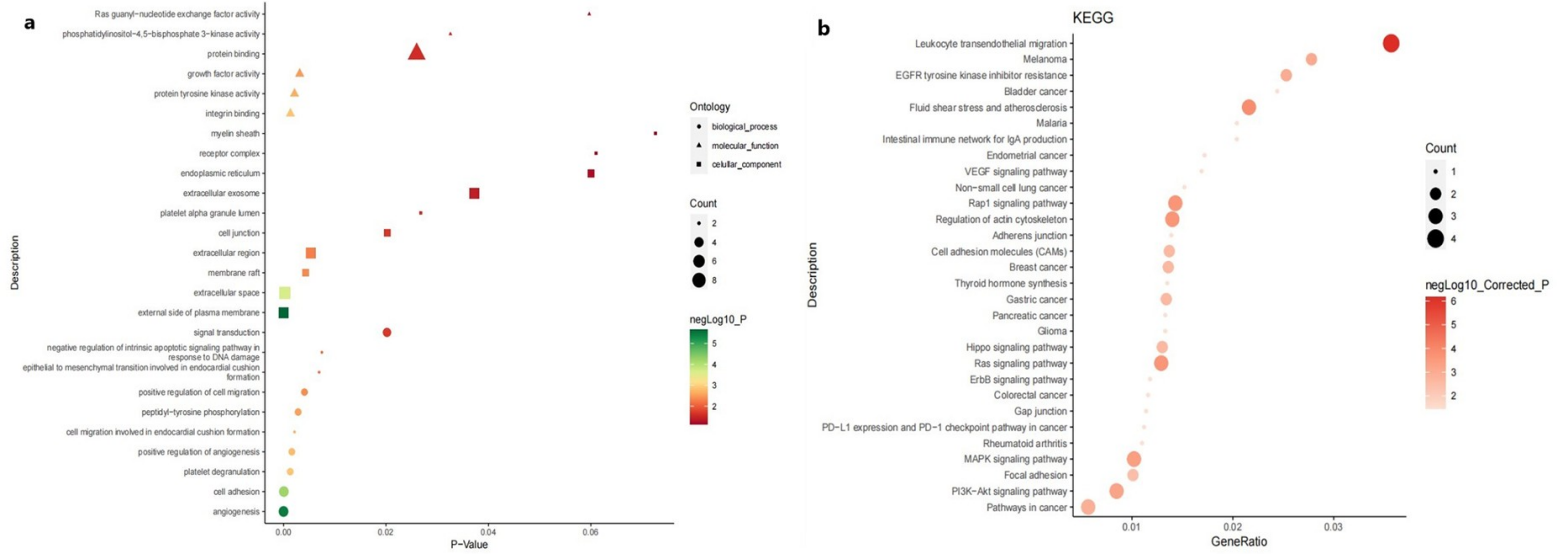
GO enrichment analysis (a) and KEGG analysis (b) of the genes in the most significant module in PRCC.

The PPI network of the DEGs was constructed with STRING and Cytoscape, and the most significant module, which consisted of 10 nodes and 43 edges, was identified (Fig 1B and 1C). DAVID and KOBAS 3.0 were used to analyse the genes in this module by functional enrichment. The results showed that the genes in this module were mainly enriched in angiogenesis, cell adhesion, platelet degranulation, leukocyte transendothelial migration, Pathways in cancer, PI3K-Akt signaling pathway and so on (Fig 3A and 3B).

### Selection and analysis of hub genes

**Fig 4:**
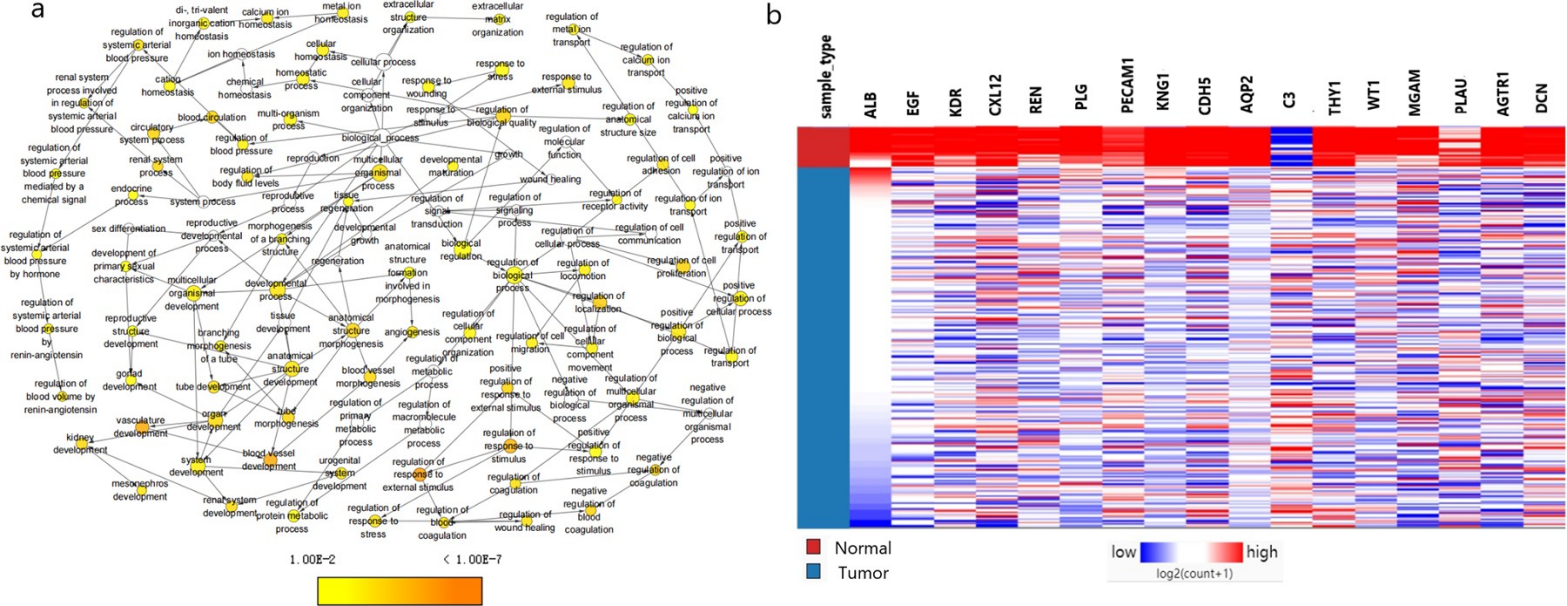
Biological process analysis and heatmap of the hub genes. (a) Biological process analysis of hub genes was performed using BiNGO. The colour depth of the nodes represents the corrected P value of the ontology. The size of the nodes represents the numbers of genes that are involved in the ontology. P<0.01 was considered statistically significant. (b) A heatmap of hub gene expression was constructed using the UCSC online database. Normal samples are shown in red, and PRCC samples are shown in blue. Red represents upregulated genes, and blue represents downregulated genes.

**Fig 5:**
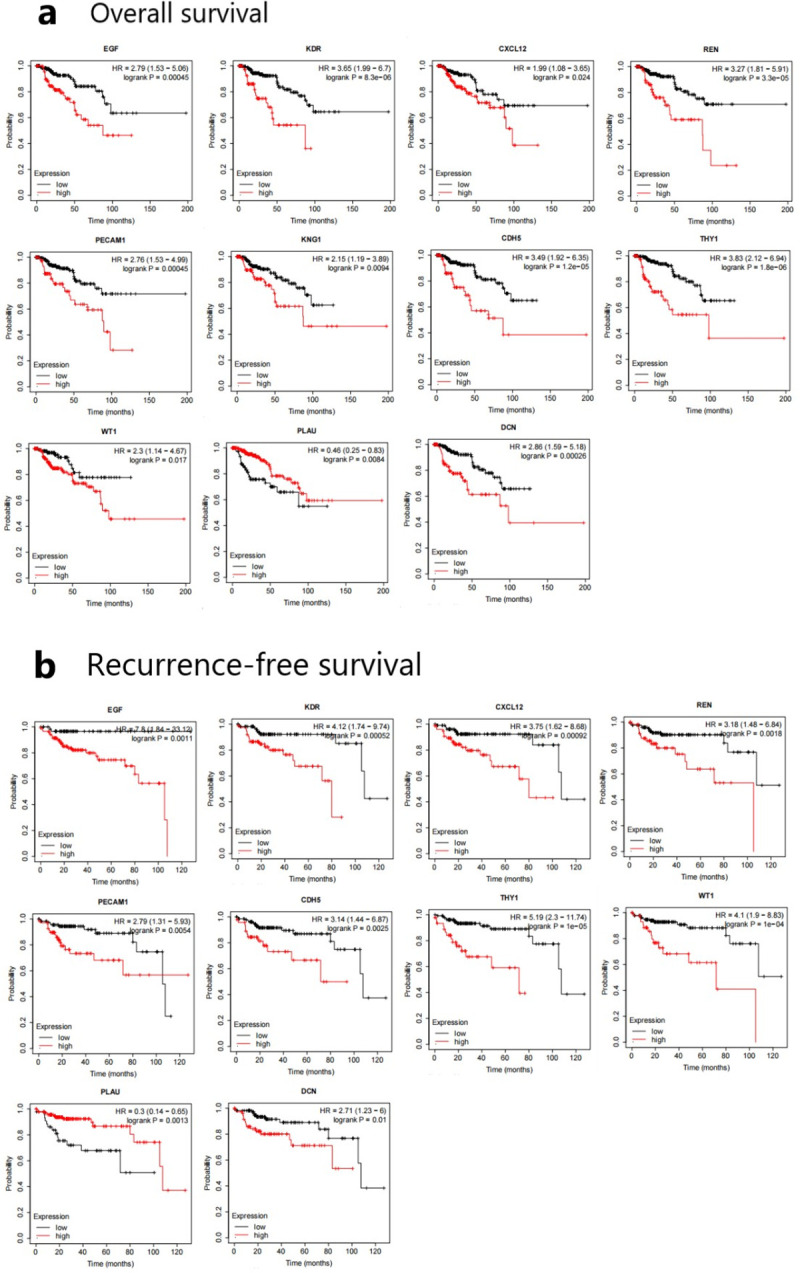
(a) Overall survival and (b) recurrence-free survival analyses of patients stratified by the expression levels of the hub genes in PRCC samples and normal samples were performed using Kaplan-Meier Plotter. P<0.05 was considered statistically significant.

**Fig 6:**
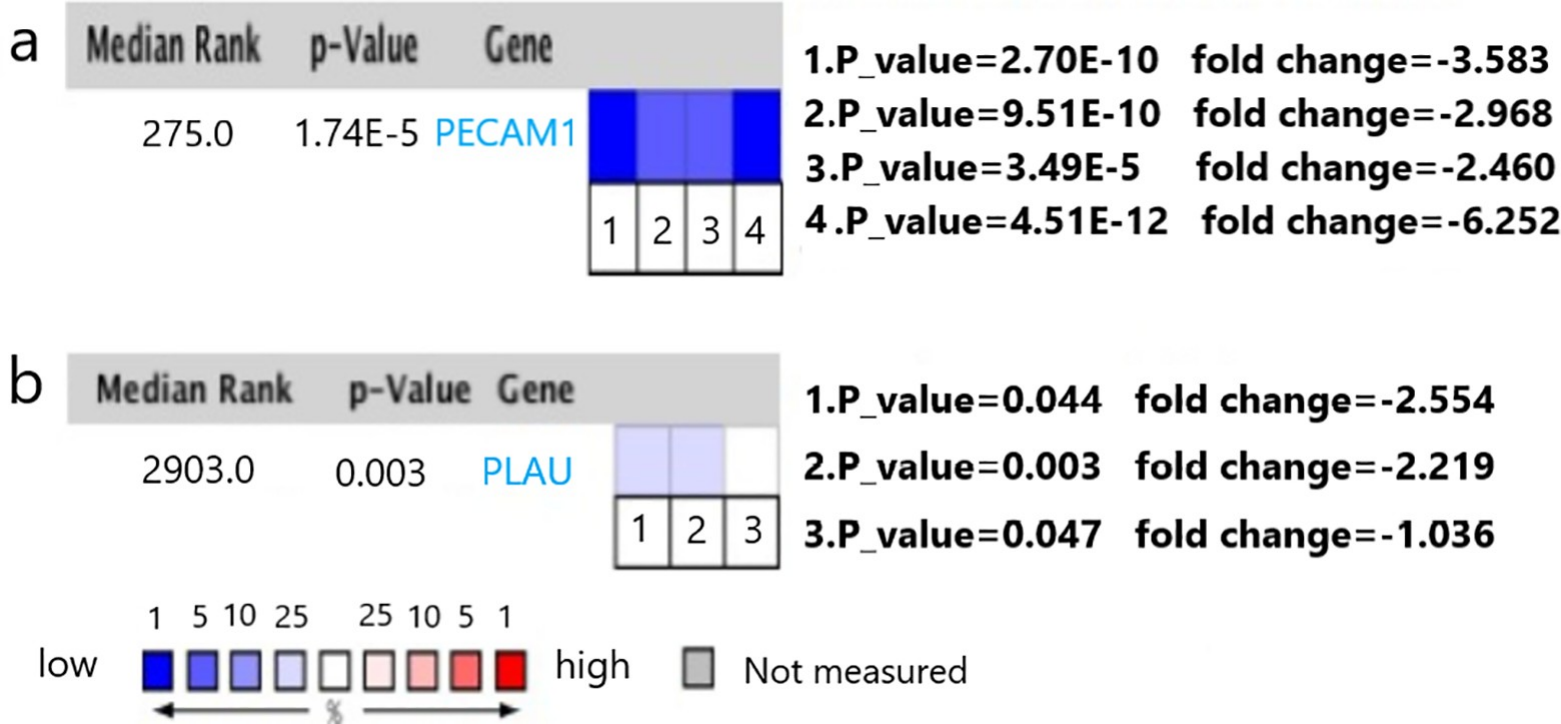
Oncomine analysis of (a) PECAM1 and (b) PLAU expression in PRCC samples vs. normal samples. Heat maps of PECAM1 and PLAU gene expression in clinical PRCC samples vs. normal samples. Red represents high expression, and blue represents low expression.

**Fig 7:**
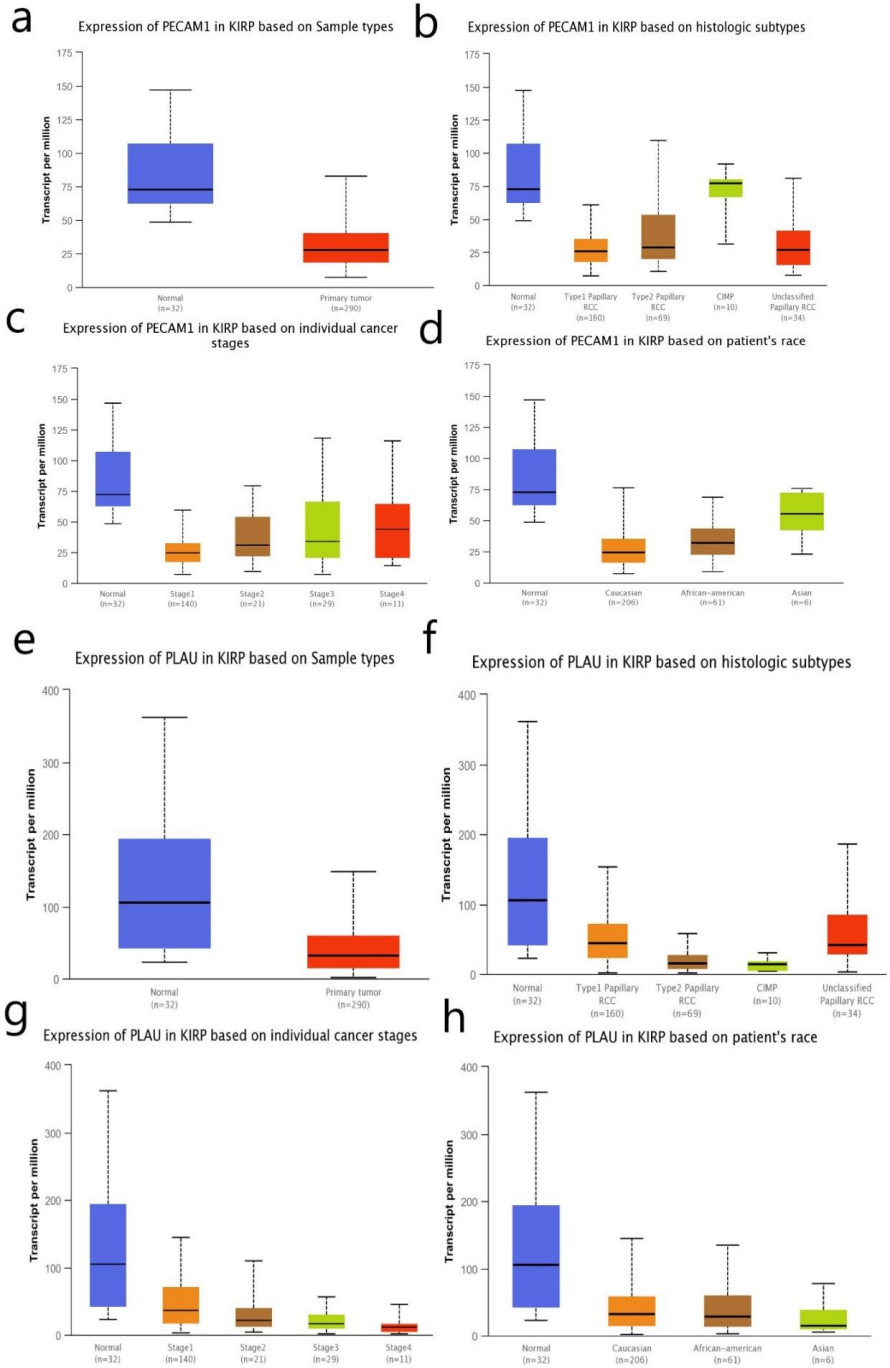
The online database UALCAN was applied to analyse the expression of the two indicated genes based on tissue type (PRCC tissue versus normal tissue), stage, subtype and race. a-d show data for PECAM1, and e-h show data for PLAU.

The 17 genes whose degree scores were greater than 15 were defined as the hub genes and included ALB, EGF, KDR, CXCL12, REN, PLG, PECAM1, KNG1, CDH5, AQP2, C3, THY1, WT1, MGAM, PLAU, AGTR1, and DCN. The results of biological process analysis of the hub genes are shown in Fig 4A. Heatmap analysis showed that the hub genes could distinguish PRCC samples from normal samples (Fig 4B). The associations of the hub genes with overall survival and recurrence-free survival were analysed using Kaplan-Meier Plotter. PRCC patients with changes in EGF, KDR, CXCL12, REN, PECAM1, CDH5, THY1, WT1, PLAU, and DCN showed poorer overall survival and recurrence-free survival. However, patients with altered KNG1 expression in PRCC tissues had poor overall survival but no significant difference in recurrence-free survival (Fig 5). Among these genes related to significant differences in overall survival and recurrence-free survival, PECAM1 and PLAU were identified as seed genes in the significant modules screened by MCODE, suggesting that they might play a crucial role in the carcinogenesis and progression of PRCC. Oncomine analysis of the carcinogenesis of PRCC and normal tissues showed that PECAM1 and PLAU were downregulated in PRCC tissues in different datasets (Fig 6). UALCAN was used to analyse the expression of these two genes based on tissue type (PRCC tissue versus normal tissue), stage, subtype and race. As shown in Fig 7, PECAM1 and PLAU were downregulated in PRCC tissue compared with normal kidney tissue, and this finding was consistent with the UCSC and Oncomine analysis results. Moreover, the expression of both genes in PRCC carcinoma tissues was related to stage, subtype and race. Notably, PECAM1 was most significantly downregulated in stage 1 Caucasian patients with type 1 PRCC, while PLAU was most significantly downregulated in Asian patients with stage 4 CIMP-type PRCC.

## Discussion

The pathological classification of papillary renal cell carcinoma is controversial, and its morbidity and mortality are increasing annually. However, the potential molecular pathogenesis of PRCC is poorly understood. Therefore, in this paper, three mRNA datasets from PRCC tissues and normal tissues were analysed. A total of 214 DEGs were identified, specifically, 205 downregulated genes and 9 upregulated genes.

GO enrichment analysis showed that the DEGs were mainly enriched in excretion, renal development, angiogenesis, negative regulation of growth, epithelial cell differentiation and other processes and were located in exosomes and other sites. By PPI network analysis, 17 hub genes were identified. Survival analysis of patients stratified by the expression levels of the hub genes showed that changes in EGF, KDR, CXCL12, REN, PECAM1, CDH5, THY1, WT1, PLAU and DCN were associated with overall survival and recurrence-free survival. These results suggest that these genes may play an important role in the carcinogenesis, progression and recurrence of PRCC. EGF can activate several pro-oncogenic intracellular pathways leading to tumour cell proliferation and angiogenesis [6]. KDR is a specific receptor for VEGF, which promotes angiogenesis [7]. CXCL12 has been shown to be a stimulatory chemokine related to tumour neovascularization and to be involved in promoting the migration and invasion of cancer cells [8]. The renin-angiotensin system regulates angiogenesis, cell differentiation and proliferation, opening a new avenue for understanding the occurrence of renal carcinoma [9]. PECAM1 encodes a protein associated with angiogenesis and extracellular circulation that is involved in tumour growth and spread [10]. CDH5 is abnormally expressed in a variety of human cancers and plays an important role in angiogenesis [11]. THY-1 is a renal differentiation marker and is useful in the characterization of tumours of renal origin [12]. The WT1 gene, which encodes a transcription factor with four zinc finger DNA-binding motifs, shows variable stage-specific expression in the developing kidney [13]. PLAU encodes a serine protease involved in degradation of the extracellular matrix and possibly in tumour cell migration and proliferation [14]. DCN is an important component of the ECM and functions as a tumour suppressor in RCC that can inhibit the proliferation and metastasis of RCC cells [15]. The genes analysed above may provide a new direction for further studies on the pathogenesis of PRCC.

KEGG pathway analysis showed that the DEGs were mainly concentrated in metabolic pathways, pathways in cancer, complement and coagulation cascades, proteoglycans in cancer and the PPAR signalling pathway. Studies have shown that metabolic reprogramming of PRCC is associated with upregulation of many proteins and enzymes involved in the glycolytic pathway [16]. PPARα antagonism inhibits several pivotal cancer-relevant metabolic pathways, leading to reduced tumour growth in RCC [17]. Further study of these potential pathways will contribute to a deeper understanding of PRCC.

PECAM1 and PLAU were identified as seed genes in the significant module determined by MCODE. Studies have shown that PECAM1 expression is significantly elevated in patients with ccRCC in the early stage of the disease [18]. However, our analysis showed that PECAM1 expression was downregulated in patients with stage 1 type 1 PRCC. Therefore, PECAM1 may be an early diagnostic indicator to distinguish ccRCC from PRCC. UALCAN analysis showed that low expression of PECAM1 and PLAU in PRCC tissues was associated with stage, subtype, and race. Notably, PECAM1 was most significantly downregulated in Caucasian patients with stage 1 type 1 PRCC, while PLAU was most significantly downregulated in Asian patients with stage 4 CIMP-type PRCC. These results suggest that PECAM1 and PLAU play critical roles in the typing and the efficient diagnosis and treatment of PRCC.

In summary, a bioinformatics approach was used in this study to analyse the DEGs involved in the occurrence and development of PRCC. Due to the current limited understanding of these DEGs, their specific biological functions and molecular regulatory mechanisms still need to be confirmed by further studies.

## Supporting information

S1 Fig Volcano plot of DEGs in three datasets. Red represents upregulation, green represents downregulation, and grey represents no significant difference. A |log_2_FC (fold change) |> 1 and adjusted P value <0.05 were considered to indicate statistically significant differential expression.
(DOCX)

## References

[1] Akhtar M, Al-Bozom IA, Al Hussain T. Papillary Renal Cell Carcinoma (PRCC): An Update. Adv Anat Pathol.2019;26(2):124–132. doi: 10.1097/PAP.0000000000000220 30507616

[2] Cancer Genome Atlas Research Network, Linehan WM, Spellman PT, Ricketts CJ, Creighton CJ, Fei SS, et al. Comprehensive Molecular Characterization of Papillary Renal-Cell Carcinoma. N Engl J Med.2016;374(2):135–45. doi: 10.1056/NEJMoa1505917 26536169 PMC4775252

[3] Toro JR, Nickerson ML, Wei MH, Warren MB, Glenn GM, Turner ML, et al. Mutations in the fumarate hydratase gene cause hereditary leiomyomatosis and renal cell cancer in families in North America. Am J Hum Genet.2003;73(1):95–106. doi: 10.1086/376435 12772087 PMC1180594

[4] Furge KA, Chen J, Koeman J, Swiatek P, Dykema K, Lucin K, et al. Detection of DNA copy number changes and oncogenic signaling abnormalities from gene expression data reveals MYC activation in high-grade papillary renal cell carcinoma. Cancer Res.2007;67(7):3171–6. doi: 10.1158/0008-5472.CAN-06-4571 17409424

[5] Jones J, Otu H, Spentzos D, Kolia S, Inan M, Beecken WD, et al. Gene signatures of progression and metastasis in renal cell cancer. Clin Cancer Res.2005;11(16):5730–9. doi: 10.1158/1078-0432.CCR-04-2225 16115910

[6] Teixeira AL, Dias F, Ferreira M, Gomes M, Santos JI, Lobo F, et al. Combined Influence of EGF+61G>A and TGFB+869T>C Functional Polymorphisms in Renal Cell Carcinoma Progression and Overall Survival: The Link to Plasma Circulating MiR-7 and MiR-221/222 Expression. PLoS One. 2015;10(4):e0103258. doi: 10.1371/journal.pone.0103258 25909813 PMC4409046

[7] del Puerto-Nevado L, Rojo F, Zazo S, Caramés C, Rubio G, Vega R, et al. Active angiogenesis in metastatic renal cell carcinoma predicts clinical benefit to sunitinib-based therapy. Br J Cancer. 2014;110(11):2700–7. doi: 10.1038/bjc.2014.225 24786599 PMC4037833

[8] Janssens R, Struyf S, Proost P. Pathological roles of the homeostatic chemokine CXCL12. Cytokine Growth Factor Rev. 2018;44:51–68. doi: 10.1016/j.cytogfr.2018.10.004 30396776

[9] Errarte P, Beitia M, Perez I, Manterola L, Lawrie CH, Solano-Iturri JD, et al. Expression and activity of angiotensin-regulating enzymes is associated with prognostic outcome in clear cell renal cell carcinoma patients. PLoS One.2017;12(8):e0181711. doi: 10.1371/journal.pone.0181711 28809959 PMC5557356

[10] He Y, Liu Z, Qiao C, Xu M, Yu J, Li G. Expression and significance of Wnt signaling components and their target genes in breast carcinoma. Mol Med Rep.2014;9(1):137–43. doi: 10.3892/mmr.2013.1774 24190141

[11] Wang Y, Zhou X, Han P, Lu Y, Zhong X, Yang Y, et al. Inverse correlation of miR-27a-3p and CDH5 expression serves as a diagnostic biomarker of proliferation and metastasis of clear cell renal carcinoma. Pathol Res Pract.2021;220:153393. doi: 10.1016/j.prp.2021.153393 33740544

[12] Hazen-Martin DJ, Chao CC, Wang IY, Sens DA, Garvin AJ, Wang AC. Developmental pattern of Thy-1 immunoreactivity in the human kidney and the application to pediatric renal neoplasms. Pediatr Pathol. 1993;13(1):37–52. doi: 10.3109/15513819309048191 8097308

[13] Szponar A, Kovacs G. Expression of KRT7 and WT1 differentiates precursor lesions of Wilms’ tumours from those of papillary renal cell tumours and mucinous tubular and spindle cell carcinomas. Virchows Arch.2012;460(4):423–7. doi: 10.1007/s00428-012-1209-z 22382985

[14] Lin M, Zhang Z, Gao M, Yu H, Sheng H, Huang J. MicroRNA-193a-3p suppresses the colorectal cancer cell proliferation and progression through downregulating the PLAU expression. Cancer Manag Res.2019;11:5353–5363. doi: 10.2147/CMAR.S208233 31354344 PMC6578599

[15] Xu Y, Xia Q, Rao Q, Shi S, Shi Q, Ma H, et al. DCN deficiency promotes renal cell carcinoma growth and metastasis through downregulation of P21 and E-cadherin. Tumour Biol.2016; 37(4):5171–83. doi: 10.1007/s13277-015-4160-1 26547587

[16] Almeida LMCA, Silva R, Cavadas B, Lima J, Pereira L, Soares P, et al. GLUT1, MCT1/4 and CD147 overexpression supports the metabolic reprogramming in papillary renal cell carcinoma. Histol Histopathol. 2017;32(10):1029–1040. doi: 10.14670/HH-11-863 28028797

[17] Abu Aboud O, Donohoe D, Bultman S, Fitch M, Riiff T, Hellerstein M, et al. PPARα inhibition modulates multiple reprogrammed metabolic pathways in kidney cancer and attenuates tumor growth. Am J Physiol Cell Physiol.2015;308(11):C890–8. doi: 10.1152/ajpcell.00322.2014 25810260 PMC4451352

[18] Yang JF, Shi SN, Xu WH, Qiu YH, Zheng JZ, Yu K, et al. Screening, identification and validation of CCND1 and PECAM1/CD31 for predicting prognosis in renal cell carcinoma patients. Aging (Albany NY). 2019;11(24):12057–12079. doi: 10.18632/aging.102540 31850854 PMC6949065

