# Supplementary material for: Screening and identification of key biomarkers of papillary renal cell carcinoma by bioinformatic analysis
Source: PLoS One. 2021 Aug 6;16(8):e0254868. doi: 10.1371/journal.pone.0254868 (PMC8345835; doi:10.1371/journal.pone.0254868)
Supplement: S1 Fig — Red represents upregulation, green represents downregulation, and grey represents no significant difference. A |log2FC (fold change) |> 1 and adjusted P value <0.05 were considered to indicate statistically significant differential expression. (DOCX) [file pone.0254868.s001.docx]

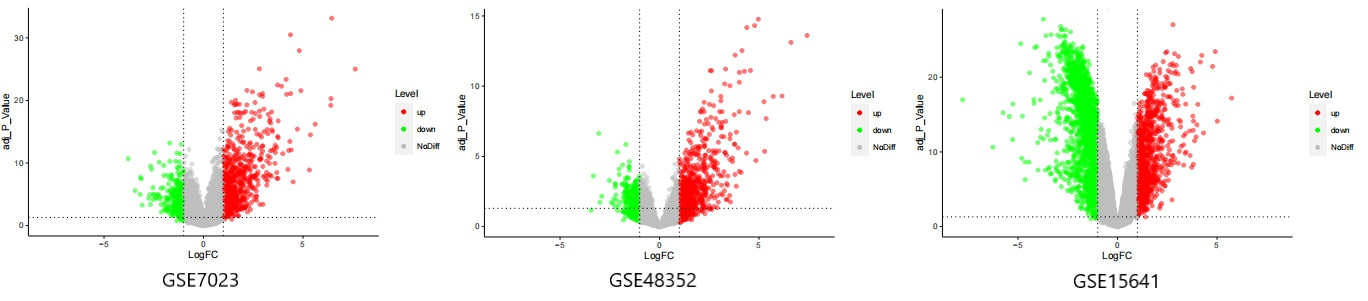


**S1 Fig. Volcano Plot of DEGs in three datasets.** Red represents up-regulated, green represents down-regulated, and gray represents no significant difference. |log_2_FC (fold change) |> 1 and adjusted P value <0.05 were considered statistically significant.
